# Clothing air gaps in various postures in firefighters’ work

**DOI:** 10.1007/s00484-022-02391-2

**Published:** 2022-11-03

**Authors:** Agnes Psikuta, Fawzy Sherif, Emel Mert, Sumit Mandal, Simon Annaheim

**Affiliations:** 1 Laboratory for Biomimetic Membranes and Textiles, Empa, Lerchenfeldstrasse 5, 9014 St. Gallen, Switzerland; 2 Department of Clothing and Textiles, Faculty of Home Economics, Menoufia University, Shibin Al Kawm, Egypt; 3 Department of Design, Housing and Merchandising, Oklahoma State University, Stillwater, OK USA

**Keywords:** Firefighter clothing, Air gap, Body posture, Thermal insulation

## Abstract

Both the physical properties of the fabric materials used in clothing and the effective design of the clothing, primarily in terms of the air gap thickness, restrict the transmission of the thermal energy from the heat source to the firefighter’s body. The air gap distribution over the body in real deployment conditions of firefighters will vary, and is likely to be different from the air gap distribution in standardised manikin tests in standing upright posture. In this study, we investigated differences in the distribution of air layers in firefighters' clothing in three postures reflecting realistic on-duty exposure conditions (crawling, hose-holding, and standing upright used in laboratory tests) using 3D body scanning technology. The body posture induced substantial changes in the air gap thickness on the upper body (chest and back) and lower body. These changes were reflected in both the thermal and evaporative resistance of the ensemble, and consequently, in their potential thermal performance in the field. Therefore, it is recommended to consider body postures during the evaluation of clothing protective performance. Secondly, the knowledge of local clothing properties in real-life exposure provides a true protection mapping and gives design inputs to improve the local protective properties of firefighters' clothing.

## Introduction

The protective performance of firefighter clothing is an important issue in clothing research as the health and safety of firefighters depend on it. Despite the development of new and better protecting materials, still many burn injuries occur during firefighting worldwide (US Fire Administration 2020). To address this issue, different aspects of firefighter clothing design have been reviewed as contributors to energy transfer through the clothing. It has been found that both the physical properties of the fabric materials used in the clothing and the effective design of the clothing could restrict the transmission of the thermal energy from the heat source to the firefighter’s body. For example, Behnke et al. (1992), Crown et al. (1993), and Schmid et al. (2016) found that the properties of the fabrics such as weight, thickness, and thermal resistance could restrict the thermal energy transmission. Additionally, research showed that the effective design of the clothing related to cuff/collar closures, zippers, and/or pockets could negatively or positively affect the local protective performance of clothing (Pawar 1995; Crown et al. 1998; Mah and Song 2010a, 2010b; Rossi et al. 2014; Song 2007). Furthermore, several studies claimed that restricting the transmission of this thermal energy from the clothing to the skin primarily depends on the existence of air gaps within and underneath the firefighter's clothing (Fu et al. 2014; Mah and Song 2010a).

The thermal performance of firefighter ensembles is typically assessed in laboratory tests. Benchmark tests, such as the hot plate for thermal and evaporative resistance and TPP test for prediction of burn injury, focus predominantly on the properties of the fabric assembly alone. Some pioneering studies included some homogeneous air layers in the measurement setup to mimic better the real exposure in clothing (Fu et al. 2014; McQuerry et al. 2018; Psikuta et al. 2018a, 2018b; Mandal et al. 2014). Furthermore, the experimental studies were extended by simulations that proved the lower protective performance of heterogeneous air gaps, which are more likely to occur in clothing than homogeneous air gaps (Deng et al. 2021). Nonetheless, the realistic thermal performance of firefighter clothing can only be measured using full body manikins, such as thermal sweating manikins or flame manikins, where the thermal conditions at the skin and clothing surface can be faithfully reproduced. For example, the thermal plume around the body and free convection inside the air layers underneath the clothing are dependent on the geometry size and shape, and are not reproducible in benchmark tests. Although full body manikins have more realistic geometry, they are used in one standard body posture — standing upright (ISO15831:^2004^; ISO13506-1:^2017^; ASTM F1291-16; ASTM F2370-16). To characterise the realistic protective performance of firefighter clothing, the distribution of the air layers and their thickness in actual working postures need to be known. So far, only one study has tackled the issue of air layer distribution in different body postures including typical working postures such as assembly work, driving, or office work (Mert et al. 2017), but it did not include postures typical for the firefighting profession. In real-life scenarios, firefighters are typically exposed in different postures to high radiant heat while firefighting or to flash-over situations in emergency. In structure search-and-rescue actions, the firefighters need to crawl to stay in layers of cooler air with lower smoke concentration. In structure and wildland firefighting, they will tend to kneel and bend down to resist the forces from the hose and to reduce the body surface area exposed to high radiant heat from the fire. The air gap distribution over the body will obviously vary between these postures, and is likely to be different from the air gap distribution in standardised manikin tests in standing upright posture (ISO13506-1:^2017^). Ultimately, these differences in the air gap magnitude may have an impact on the protective performance of the clothing.

This study aims to investigate the distribution of the air gap thickness in firefighters' protective clothing in different critical working body postures, such as crawling and hose-holding, compared to the typical standing upright posture used in laboratory studies. For this purpose, an agile shop window mannequin, an exemplary firefighter ensemble, 3D scanner, and the refined evaluation methodology developed in earlier studies were applied to determine the local air gap distribution. Comparison of the air gap distribution in three postures will help to understand the differences in the protective performance of the clothing in different exposure cases of on-duty firefighters and support the development of a new approach to the design of firefighter clothing.

## Methodology

### Study design to evaluate air gaps in various postures

Three body postures were analysed in this study — one representing a typical body posture in manikin measurements — standing upright, and two relevant to firefighters’ tasks, such as crawling and hose-holding. To evaluate the air gaps in these postures, an agile shop window mannequin was used together with dedicated stands to keep the mannequin limbs in place. A dedicated firefighter ensemble was prepared to fit the mannequin and allow for easy manipulation on the mannequin attached to the stand. A 3D scanning technique was used to collect the data of the nude and dressed mannequin in the evaluated postures, and the post-processing methodology provided the air gap volumes and air gap thickness at selected body regions.

### Agile mannequin

**Fig. 1:**
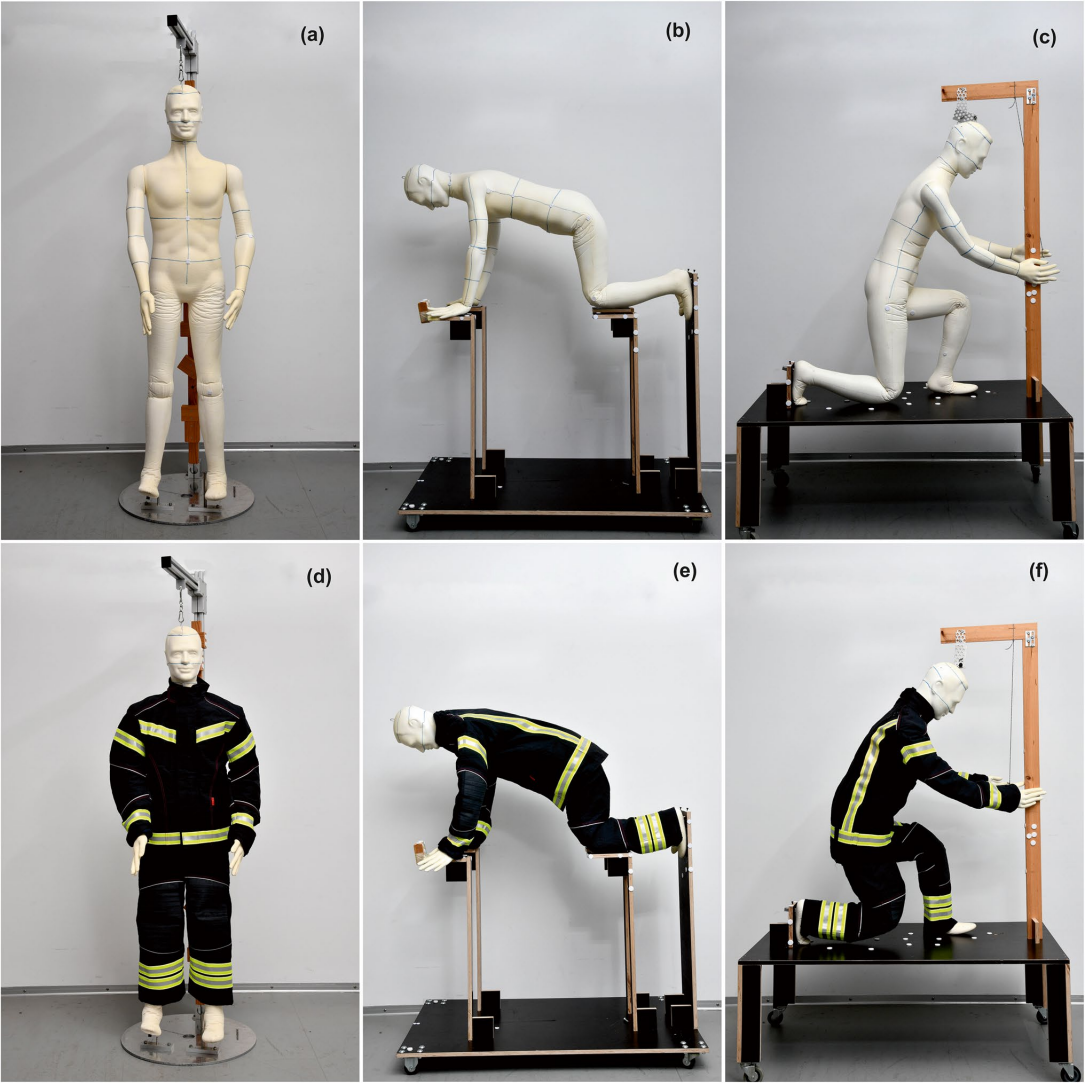
Body postures analysed in this study including standing upright (**a**, **d**), crawling (**b**, **e**), and hose-holding (**c**, **f**) demonstrated on the nude and dressed agile mannequin fixed to dedicated stands

**Fig. 2:**
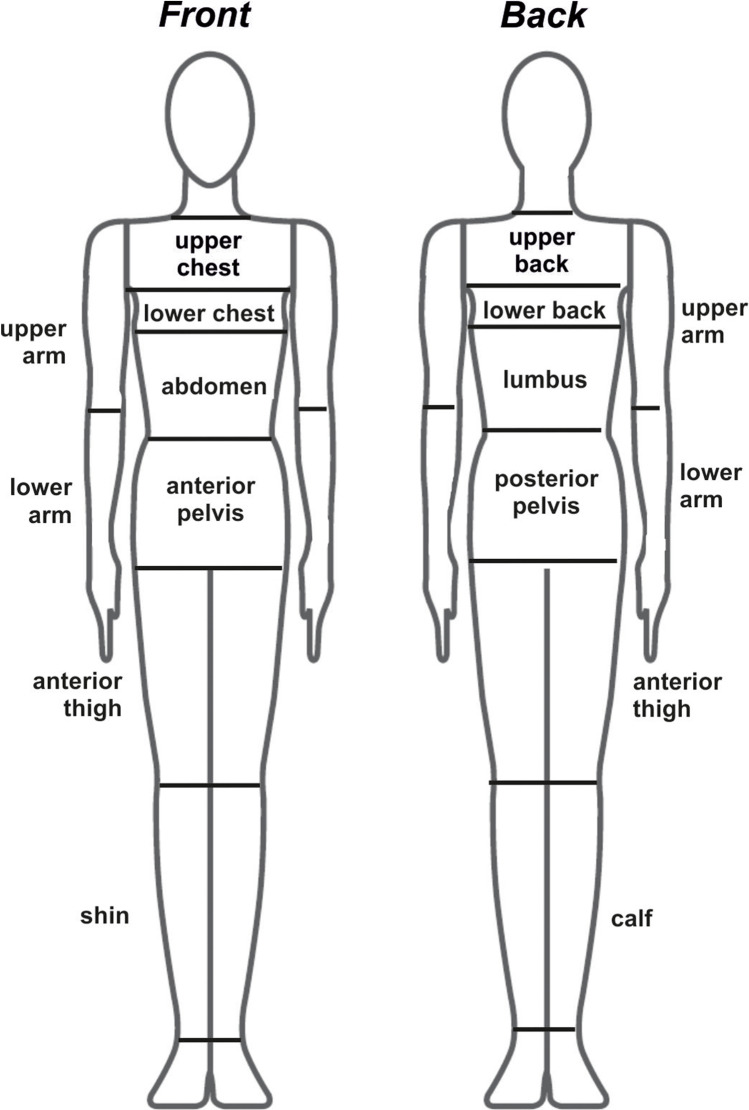
Mannequin body division into individual regions for which air gap thickness was evaluated

A whole-body agile male mannequin was used in this study (Polyform® GmbH & Co. KG., Germany) (Fig. 1). The basic body dimensions of this mannequin were 180 cm height, 91 cm girth at the chest, and 77 cm girth at the waist, all measured according to ISO 8559:^2017^ (Table 1). The mannequin surface area was divided into individual body parts used in analysis of the air gap thickness (Fig. 2). Its joints allowed for setting the mannequin to the relevant body postures of firefighters performing typical tasks, such as crawling and hose-holding (Fig. 1b and c). This was done with the help of dedicated stands fixing the joints in the desired positions (Fig. 1b and c). The standing upright posture was also used for comparison to the typical body posture used in manikin measurements (Fig. 1a).

**Table 1:** The agile mannequin body dimensions and ease allowances of firefighter ensemble in relation to respective body landmarks according to ISO 8559:^2017^ (ease allowance is the difference between mannequin and garment circumferences at given body landmarks)

Body parts (garments)	Upper body (jacket)				Lower body (trousers)		
	Chest	Waist	Hip	Upper arm (biceps)	Hip	Thigh	Lower leg
Mannequin circumferences (cm)	91	77	91	26	91	48	37
Ease allowances of the firefighter ensemble (cm)	23	39	20	15.5	11	10	17

### Firefighter ensemble

**Fig. 3:**
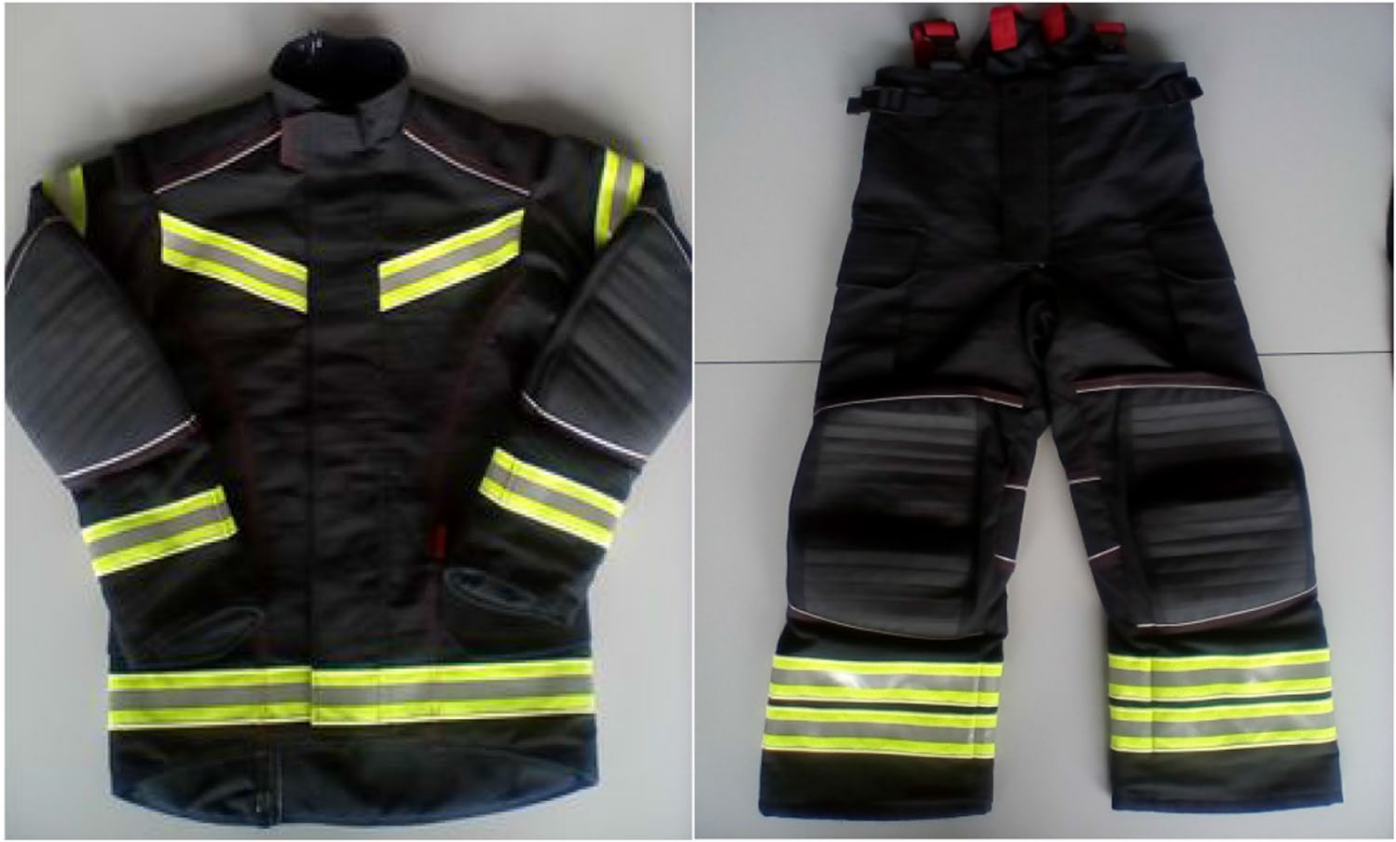
Customised firefighter clothing consisting of jacket and trousers with braces

Firefighters’ clothing consisting of a jacket and a trouser with braces (Fig. 3) was ordered in a customised size to fit the agile mannequin. Since the mannequin dimensions were less than those recommended for size S, the size XS was confectioned for this study (typically made on-demand and not for stock) (Bristol Uniform Limited, England). The ensemble fit is described by ease allowances of the garments in relation to the agile mannequin used, as described in Table 1. The multi-layered fabric assembly of the ensemble consisted of the following: meta-aramid (outer layer), PTFE-coated 25% meta-aramid/25% para-aramid/50% basophil (middle layer), and non-woven meta-aramid quilted with a 50% meta-aramid/50 FR viscose (inner layer). The fabric assembly thickness was obtained according to the ISO 5084:^1996^ standard using the thickness tester (Frank-PTI thickness tester, Germany) and approximated 3.35 mm.

### 3D scanning procedure

The 3D scanning procedure included scanning the nude and dressed mannequin in the same posture (Fig. 1). A hand-held 3D body scanner Artec MHT (Artec Group, USA) was used. The scanning process was done manually by moving the scanner around the manniquin at a defined distance band (40–90 cm) and at approximately 90° angle to the mannequin surface by a trained operator. For some postures (crawling and hose-holding), the mannequin’s bent limbs obstructed the scanning process of the neighbouring body regions and scanning had to be completed in stages. The scanned patches were subsequently aligned according to the determined common reference points, e.g., nose, ear, foot, and geometrical marks on the stand, that were scanned in every patch. The process of scanning the nude and clothed manikin for a particular posture was repeated 4 times, redressing the mannequin between the repetitions.

### Scan post-processing and calculation of local mean air gap thickness

The 3D scans of the nude and clothed mannequins were further processed in the dedicated surface inspection software Geomagic Control 2015 (3D Systems®, USA) to remove scanning artefacts and divide the body volume into regions as shown in Fig. 2. For the standing upright posture, the applied air gap determination methodology was based on previous studies (Psikuta et al. 2012; Mert et al. 2017), where the scans of nude and dressed mannequins were superimposed and the distance between surfaces calculated in post-processing software. In the case of the crawling and hose-holding postures, this methodology failed since it requires the mannequin geometry to be identical for the nude and dressed case. The firefighter ensemble used in this study deformed the mannequin slightly due to its substantial weight, and the volumetric air gap determination had to be applied. This means that the mean air gap thickness for each body region was determined based on computation of the volume of the air gap (${V}_{\mathrm{Air\ gap}}$). This was done by deriving the volumes of the nude (${V}_{\mathrm{Nude}}$) and dressed (${V}_{\mathrm{Dressed}}$) body parts from the post-processing software and subtracting them from each other considering the fabric thickness (and its volume, ${V}_{\mathrm{Fabric}}$) according to the following formulae:

1 $${{V}_{\mathrm{Air\ gap} }=V}_{\mathrm{Dressed}}- {V}_{\mathrm{Nude}}-{V}_{\mathrm{Fabric}}$$

**Fig. 4:**
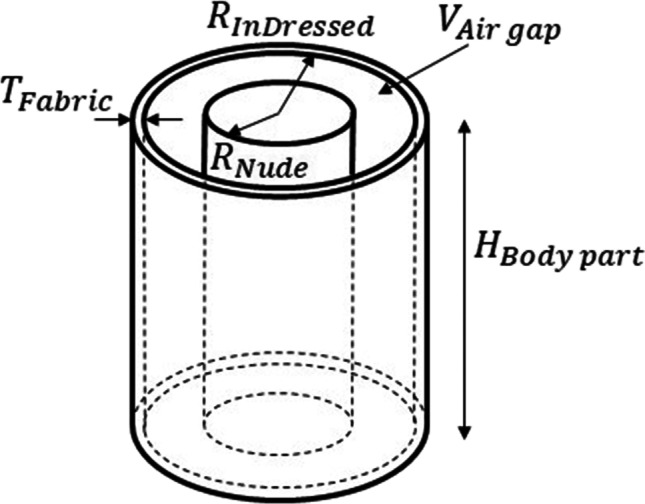
Scheme of the simplified body part geometry used for calculation of air gap thickness using volumetric method

The volume of the fabric was estimated based on the fabric thickness (${T}_{\mathrm{Fabric}}$) and outer surface area of the clothing for individual body regions derived from scans. Subsequently, the local mean air gap thickness (${AGT}_{\mathrm{Body\ part}}$) was determined based on geometrical calculation, assuming that the body part and clothing are approximated by two concentric cylinders, with the volume difference equal to ${V}_{\mathrm{Air\ gap}}$ (Fig. 4). The radius (${R}_{\mathrm{Nude}}$) of the inner cylinder representing the body part was derived from the mannequin’s local circumferences as given in Table 1, and the cylinder height (${H}_{\mathrm{Body\ part}}$) was derived based on the body part volume and the radius. The height of the outer cylinder corresponding to the clothing was assumed to be the same as that of the inner one corresponding to the body part. Finally, the air gap thickness was calculated as follows:

2 $${V}_{\mathrm{Dressed}}-{V}_{\mathrm{Fabric}}={\pi {R}_{\mathrm{InDressed}}^{2}\bullet H}_{\mathrm{Body\ part}}$$

3 $${V}_{\mathrm{Nude}}={\pi {R}_{\mathrm{Nude}}^{2}\bullet H}_{\mathrm{Body\ part}}$$

4 $${V}_{\mathrm{Dressed}}-{V}_{\mathrm{Fabric}}-{V}_{\mathrm{Nude}}={\pi H}_{\mathrm{Body\ part}}\bullet \left({R}_{\mathrm{InDressed}}^{2}-{R}_{\mathrm{Nude}}^{2}\right)$$

5 $${R}_{\mathrm{InDressed}}=\sqrt{\frac{{V}_{\mathrm{Dressed}}-{V}_{\mathrm{Fabric}}-{V}_{\mathrm{Nude}}}{\pi {H}_{\mathrm{Body\ part}}}+{R}_{\mathrm{Nude}}^{2}}$$

6 $${AGT}_{\mathrm{Body\ part}}={R}_{\mathrm{InDressed}}-{R}_{\mathrm{Nude}}$$

In the pelvis area, where the trousers and jacket overlapped, the air gap thickness between the outer layer (jacket) and the skin was computed.

### 3D scanning and post-processing accuracy determination

Based on our previous studies, the scanning and post-processing accuracy using the Artec MHT scanner and Geomagic Control software applied in the case of the standing upright posture was proved to be very good, with repeatability below 0.9 mm (Mert et al. 2017; Psikuta et al. 2015). In this study, we have also evaluated the repeatability of the volume determination by comparing the body parts volumes of four independent scans of the nude mannequin in the crawling and hose-holding postures. For both postures, the volumes of the different body parts (i.e., upper chest, lower chest, abdomen, upper back, lower back, lumbus, anterior pelvis, posterior pelvis, anterior thigh, posterior thigh, shin, calf, upper arm, lower arm) and the coefficient of variation (standard deviation divided by a mean in %) of the volumes were within the range of 0.2‒1.0%. This deviation of the volume would have a negligible effect on the air gap thickness below 0.7 mm.

### Calculation of thermal and evaporative resistance of the air gaps

The thermal end evaporative resistance of the obtained air gaps was calculated using comprehensive clothing model by Joshi et al. (2019) to demonstrate the thermal consequences of varying air gap thickness in different postures. The thermal resistance of the enclosed air layer (*R*_*ct*_, m^2^ K/W) is a composition of conductive (*h*_cond_,W/m^2^ K), convective (*h*_cov_, W/m^2^ K), and radiant (*h*_rad_, W/m^2^ K) heat transfer coefficients, which all show certain dependency on temperature in the air gap, namely:

7 $${R}_{ct}=\frac{1}{{h}_{\mathrm{cond}}+{h}_{\mathrm{conv}}+{h}_{\mathrm{rad}}}$$

8 $${h}_{\mathrm{cond}}=\frac{k}{AGT}$$

9 $${h}_{\mathrm{conv}}=Nu\ \frac{k}{AGT},\; Nu\ =f(\alpha ,\beta ,\rho ,\mu ,\vartheta )$$

10 $${h}_{\mathrm{rad}}=\frac{\sigma \left({T}_{\mathrm{inner}}^{2}+{T}_{\mathrm{outer}}^{2}\right)\left({T}_{\mathrm{inner}}+{T}_{\mathrm{outer}}\right)}{\frac{1}{{\varepsilon }_{\mathrm{inner}}}+\frac{1}{{\varepsilon }_{\mathrm{outer}}}-1}$$

where *k* (W/mK) is air thermal conductivity, *AGT* (m) is air gap thickness, *Nu* is Nusselt number, *α* (m^2^/s) is air thermal diffusivity, *β* (1/K) is thermal expansion of air, *ρ* (kg/m^3^) is air density, *μ* (kg/ms) is a dynamic air viscosity, *ν* (m^2^/s) is a kinetic air viscosity, and *ε* ( −) is emissivity of enclosing surfaces of the air gap. In Eqs. (8) and (9), *k*, *α*, *β*, *ρ*, *μ*, and *ν* are functions of air temperature in the air gap. Radiant heat transfer coefficient is directly dependent on surface temperatures enclosing the air gap (e.g., skin or underwear and inner side of firefighter protective ensemble). This implies that thermal insulation *R*_*ct*_ is also a function of temperature that may have a substantial variation within temperature range encountered in firefighter exposures. Using comprehensive clothing model by Joshi et al. (2019), the thermal resistances of air gaps obtained in this study were calculated for two cases of ambient exposure, i.e., to 20 °C and 140 °C. The first case corresponds to typical conditions in the laboratory tests dedicated measurement of clothing thermal insulation, and the latter one corresponds to rather extreme exposure to a hot environment. The inner side of the air gap was assumed to be at 34 °C (presumable skin or underwear temperature); the thermal and evaporative resistance of firefighter clothing assembly was 0.071 K m^2^/W and 16.8 Pa m^2^/W, respectively, (measured according to ISO 11092:^2014^); and the air gap thickness was taken respectively according to findings of this study. The evaporative resistance of stagnant air layer is also dependent on air temperature as the diffusion coefficient of water vapour in the air is non-linearly dependent on air temperature (Joshi 2019). In case of temperature range considered in this application (20–140 °C), the range of evaporative resistance however changes only very slightly by 0.01 Pa m^2^/W per 1 mm of air layer thickness and was further considered as a single value for all temperature gradients.

## Results

**Fig. 5:**
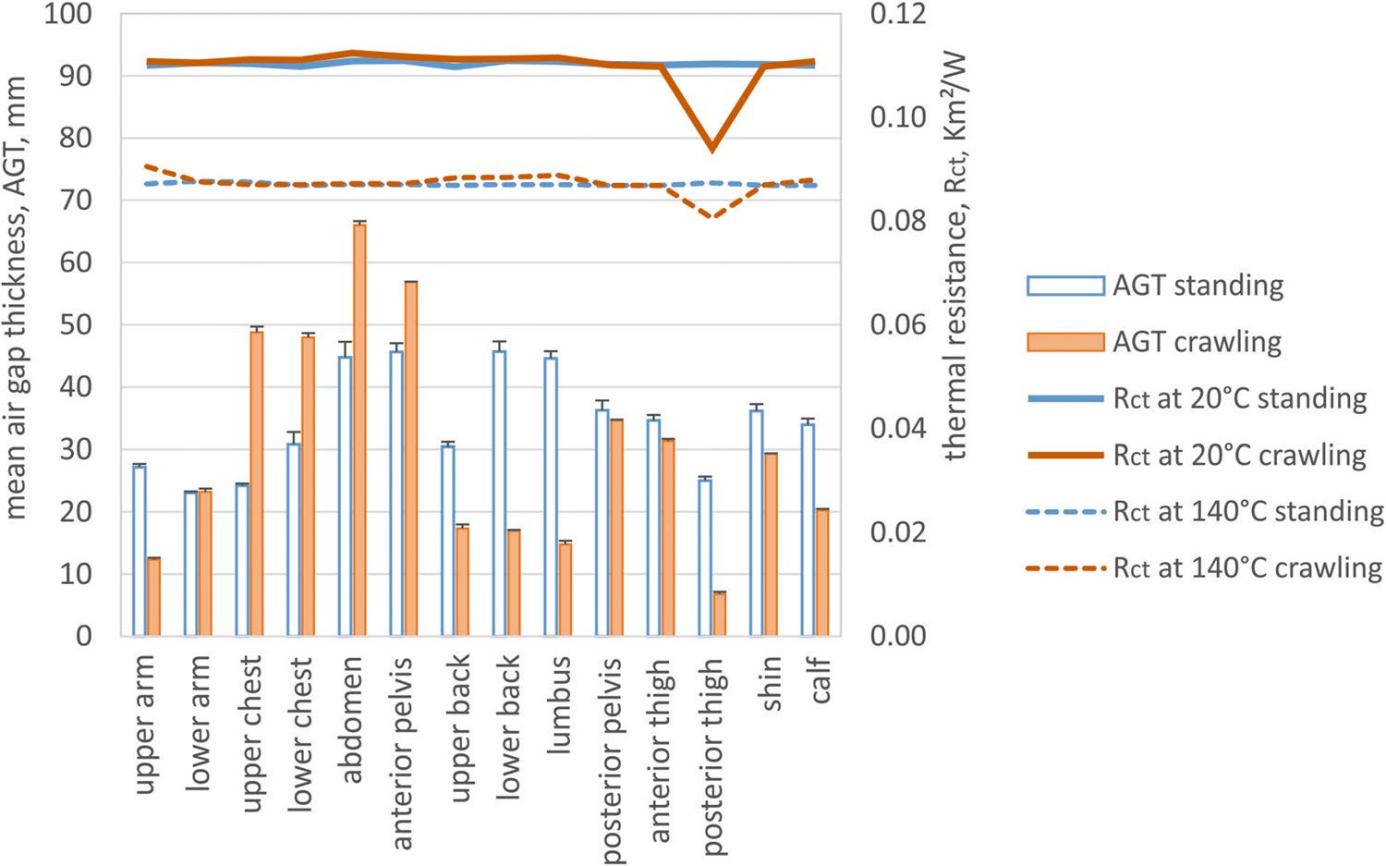
Mean local air gap thickness (AGT) and simulated thermal resistance (R_ct_) across the air gap for different temperature gradients (where T_in_ = 34 °C, T_ambient_ = 20 or 140 °C, respectively) for standing and crawling body postures (Fig. 1d and e) for individual body parts as indicated in Fig. 2

**Fig. 6:**
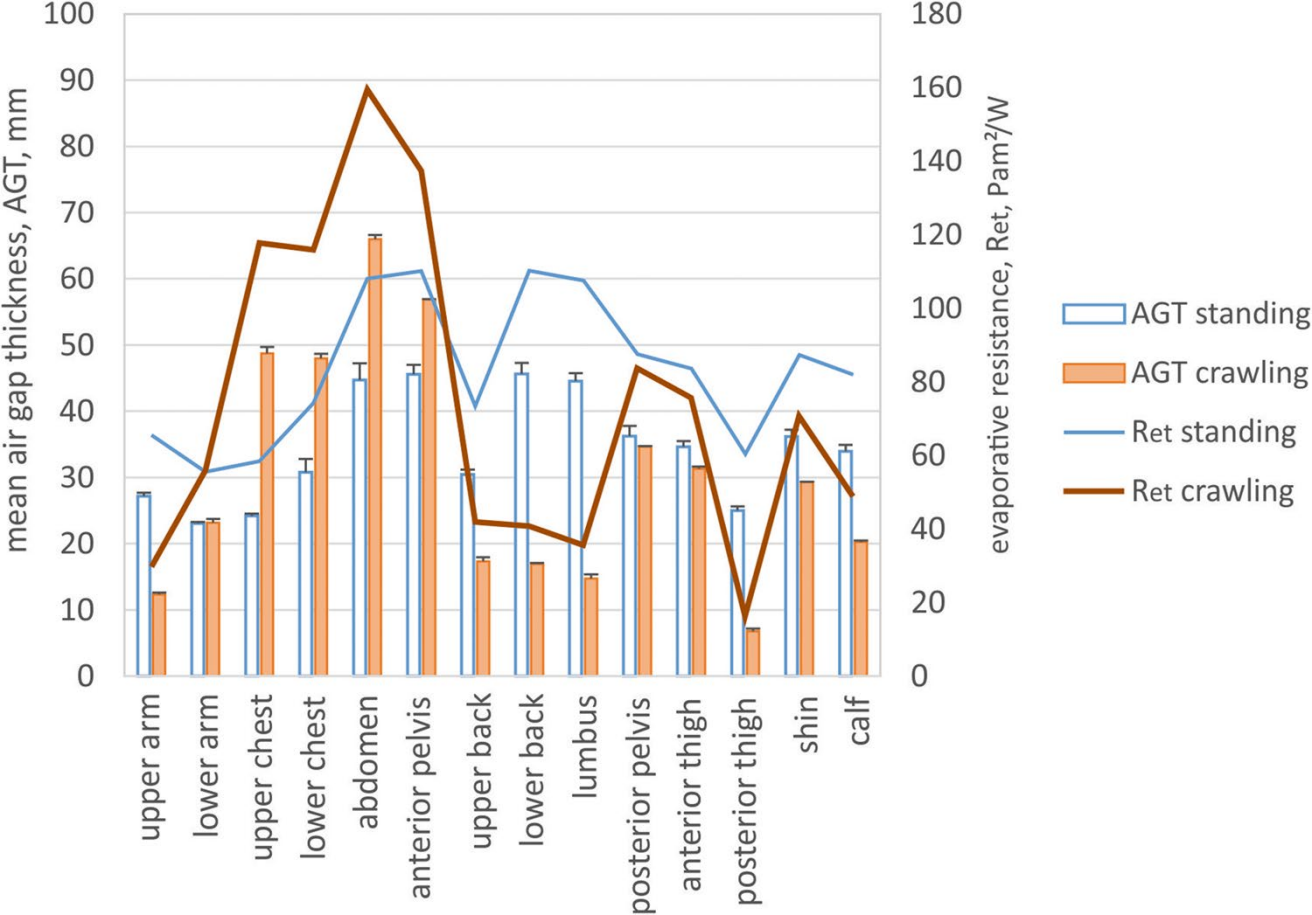
Mean local air gap thickness (AGT) and simulated evaporative resistance (R_et_) for standing and crawling body postures (Fig. 1d and e) for individual body parts as indicated in Fig. 2

**Fig. 7:**
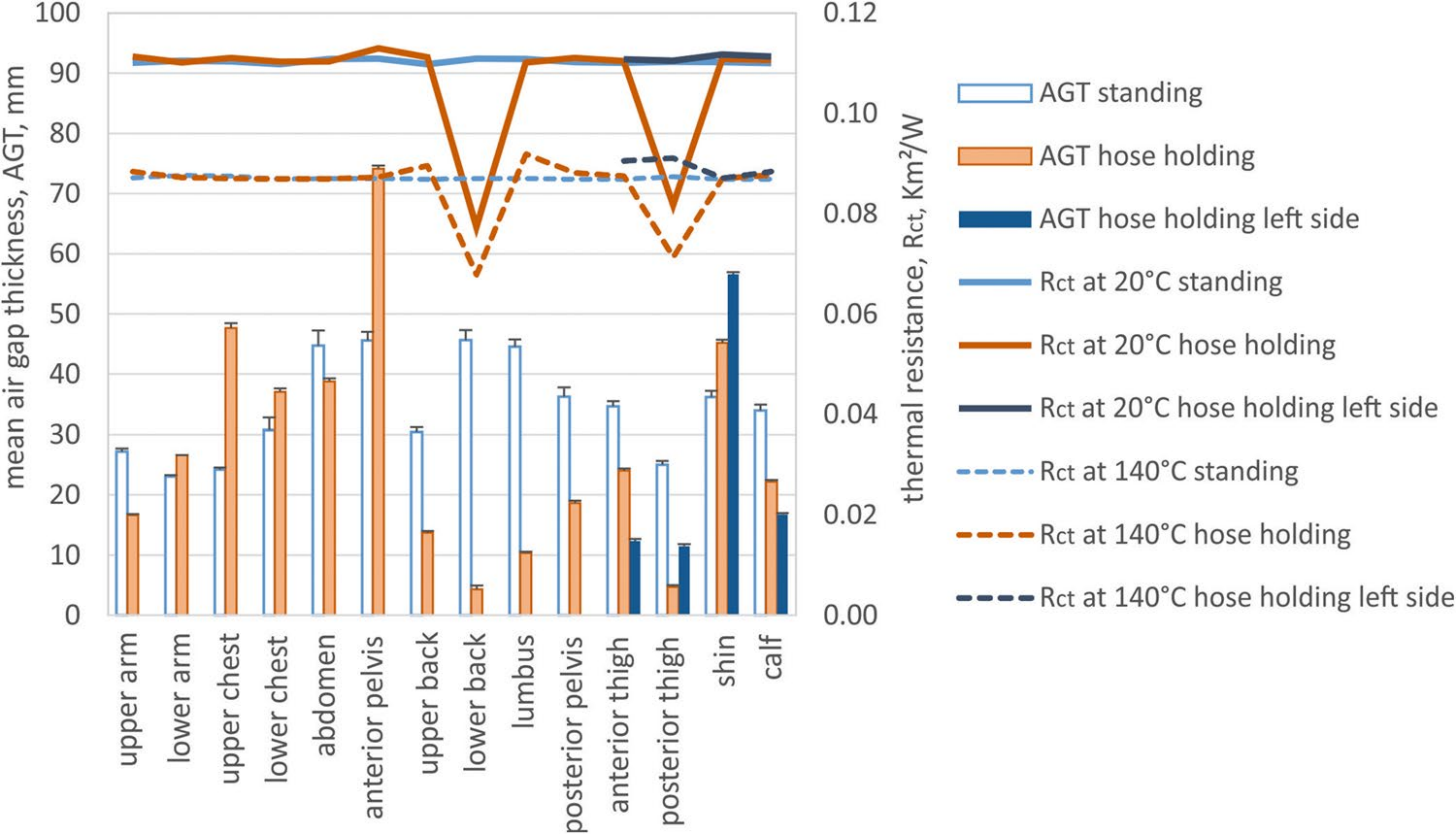
Mean local air gap thickness (AGT) and simulated thermal resistance (R_ct_) across the air gap for different temperature gradients (where T_in_ = 34 °C, T_ambient_ = 20 or 140 °C, respectively) for standing and hose-holding body postures (Fig. 1d and f) for individual body parts as indicated in Fig. 2. Since the hose-holding posture was asymmetric, both lower body sites are indicated, as the left body side with foot on the ground and the right body side with foot and knee on the ground

**Fig. 8:**
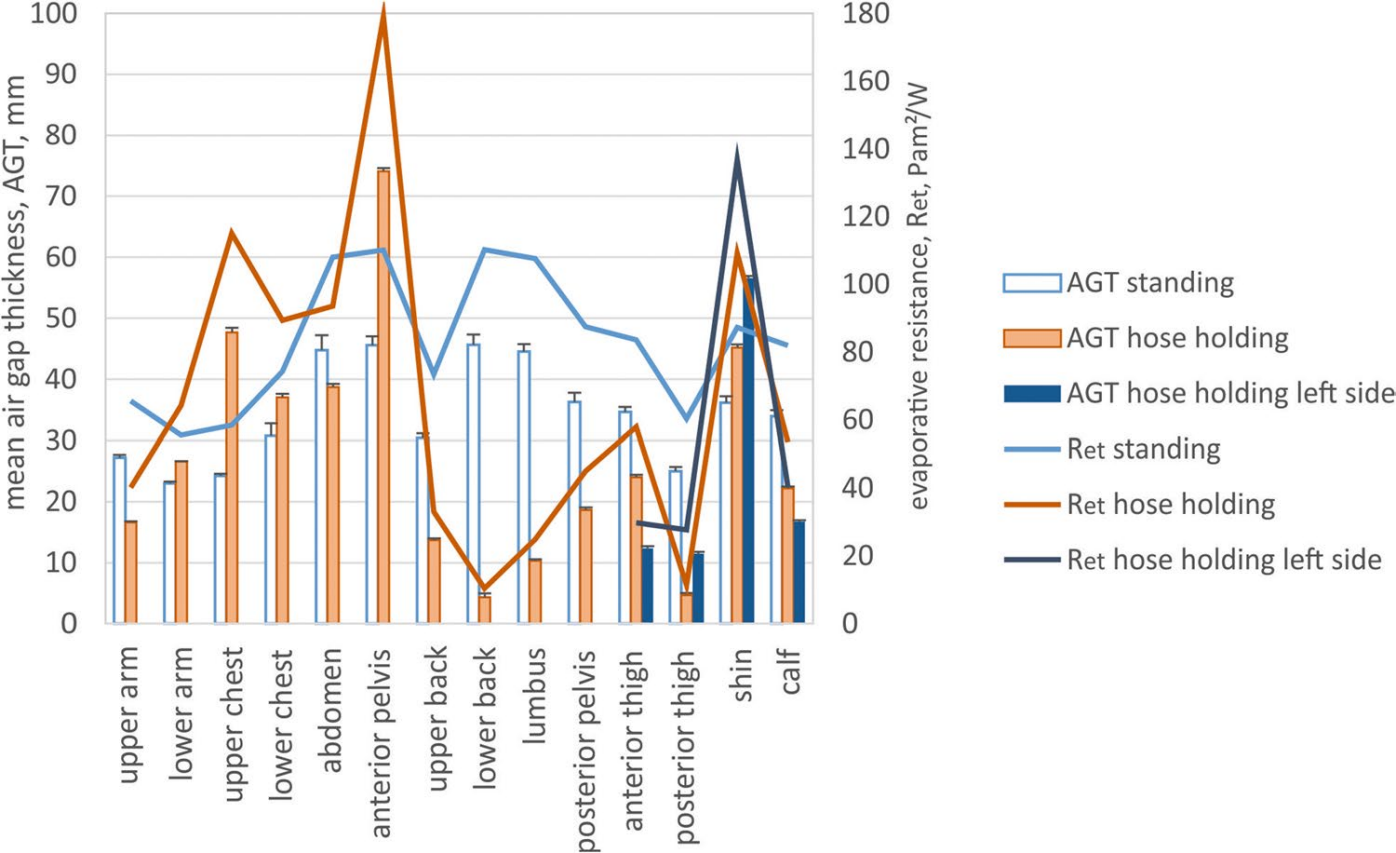
Mean local air gap thickness (AGT) and simulated evaporative resistance (R_et_) for standing and hose-holding body postures (Fig. 1d and f) for individual body parts as indicated in Fig. 2. Since the hose-holding posture was asymmetric, both lower body sites are indicated, as the left body side with foot on the ground and the right body side with foot and knee on the ground

Figures 5–8 show the air gap thickness of the individual body parts indicated in Fig. 2 during typical postures in firefighters’ work — crawling and hose-holding, respectively — in comparison to the standing upright posture typically used in measurements of clothing properties. Since the hose-holding posture was asymmetric (right leg kneeling with extended hip; left leg standing as the supporting leg for hose holding with bended hip), both lower body sites are indicated in Fig. 5. In addition, Figs. 5 and 7 show the calculated thermal resistances and Figs. 5 and 8 show the calculated evaporative resistances of obtained local air gaps using comprehensive clothing model (Joshi 2019; Joshi et al. 2019).

## Discussion

This study addressed an important issue of the distribution of air layers and their thickness in firefighter clothing during typical firefighting tasks, and compared it to their distribution in the standing upright posture used in laboratory studies to determine the protective characteristics of such clothing. Since air layers provide a substantial share of the protective properties of the clothing (Mert et al. 2015; Psikuta et al. 2018a, b), the difference between the protective performance in laboratory tests and in real-life situations may be estimated provided the air layer thickness distribution is known for both cases. In the case of the crawling body posture, the air gap thickness increased at the front torso and was substantially lower at the back torso and legs compared to the standing upright posture (Fig. 5). This is due to the gravity force forcing the jacket to hang down at the chest and belly. As the legs stretch and bend, the forces deform the garment due to limb bending, such as stretching the arms perpendicular to the torso, and bending the hip and knee joints. Effectively, the back, posterior thigh, and calf are less heat protected. This is especially relevant since this is the side of the body exposed to potentially hot surfaces, fumes, or flames. Typically, the back side is partially covered by other firefighting equipment (e.g., an oxygen cylinder with breathing apparatus in the rucksack) that can be either protective by adding insulation or detrimental by compressing the remaining air layers in adjacent regions. On the other hand, the front side of the body is better protected with its substantially larger air gaps. However, in the crawling posture, this side is directed towards the floor (which is cooler than the ceiling in fire conditions) and additionally shielded by the limbs.

In the case of the hose-holding posture, the air gap thickness in the front part of the jacket slightly increased, whereas it massively dropped at the rear part as compared to the standing posture (Fig. 7). This is a result of the limbs extending forward, which pulled the material of the jacket towards the body at the back and pushed it away from the body in front, given the substantial rigidity of the firefighter clothing. For the legs, the air gap at the thighs and calves was compressed due to leg bending at the hips and knees, which pulled the garment towards the body. At the shins, the air gap thickness increased however, as a result of the garment’s stiffness in the case of the left leg with the foot on the ground (the trouser leg wanting to stay straight despite the bended knee, resulting in the trousers leg standing out at the shin) and due to the force of gravity in the case of the right leg with foot and knee on the ground (the trouser leg hanging freely on the calf and retreating from the body surface at the shin). This air layer distribution is beneficial for more protection at the exposed front body side (increased air gap thickness) and better body heat release at the back of the body, typically at lower temperature, as it is shaded from the radiation source and has decreased air gap thickness.

**Fig. 9:**
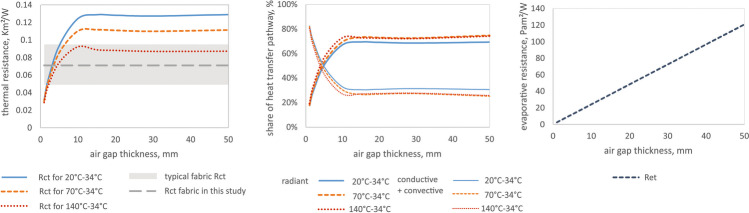
Thermal (**a**) and evaporative resistance (**c**) (R_ct_ and R_et_, respectively) and the share of the heat transfer pathway (radiant, conductive + convective) (**b**) across the air gap for different temperature gradients (where T_in_ = 34 °C, T_ambient_ = 20, 70, or 140 °C, respectively)

The thermal and evaporative resistance of the clothing is correlated with the thickness of the entrapped air layers and fabric properties; since air is a very good thermal insulator and a barrier for water vapour diffusion (the specific thermal resistance of air is 38 mK/W, and of a typical fabric 24 mK/W (Lotens and Havenith 1991), the evaporative resistance of stagnant air is 2.3 Pa m^2^/W per 1 mm of air layer thickness (Wissler and Havenith 2009)). In larger air gaps, the onset of natural convection compromises its isolating properties, but this effect is rather small (conductive and convective pathways constitute together about 30% of total heat transfer for air gaps larger than 10–15 mm; Fig. 9b) (Joshi et al. 2019). On the other hand, radiation, which is a non-linear process with regard to the temperature gradient across the air gap, is a critical heat transfer pathway in firefighter clothing (it constitutes about 70% of the total heat transfer; Fig. 9b). Being exposed to significantly higher ambient temperatures than skin temperature will result in an increased radiant heat transfer coefficient, and hence, decreased insulation of the air layers (Fig. 9a). This means that with the increasing exposure temperatures, the protective properties of the air gap will diminish, approaching values close to that of the fabric used in typical firefighters' clothing (Fig. 9a, the grey zone represents the range of thermal insulation of fabrics used in firefighters' clothing, the dashed line represents the fabric used in this study). Having a suitable clothing model (e.g., the steady-state model by Joshi et al. (2019) or transient model by Deng et al. (2021)), these properties can be computed for various temperature gradients corresponding to the possible working conditions in which this clothing could be used, including the share of different heat transfer pathways (Fig. 9a and b). The most significant thermal insulation change of the air gap is observed for thicknesses below 10–15 mm, and above that value of the thermal resistance of the air gap varies only minimally. In the standing upright posture, the air gap thickness is larger than 20 mm for all body regions, which implies rather homogeneous insulation provided by the enclosed air gap in addition to the thermal resistance of the fabric. The air gap thickness reduced below 10–15 mm for several body parts in both postures examined in this study, such as the upper arm, back regions, and especially posterior thigh in the crawling posture and the back regions and posterior and anterior thighs in the hose-holding posture. The protection of these body parts will therefore be less compared to the protection level determined in the manikin standing upright. The simulated local thermal resistances for corresponding body postures show the magnitude of the local thermal insulation loss due to the posture alteration from standing, which can be as much as 15% and 8% for posterior thigh in crawling posture, 30% and 22% for lower back, and 26% and18% for right posterior thigh in hose-holding posture for ambient temperatures of 20 °C and 140 °C, respectively (Figs. 5 and 7). The evaporative resistance is much more prone to changes in air gap thickness since it is directly proportional to its variation. In crawling posture, the local evaporative resistance changed as much as 27–201% (or between 59 Pa m^2^/W smaller and 72 Pa m^2^/W greater in absolute terms) compared to standing posture (Fig. 6). Even greater changes in evaporative resistance were observed for hose-holding posture, such as 9–197% (or between 69 Pa m^2^/W smaller and 100 Pa m^2^/W greater in absolute terms) compared to standing posture (Fig. 8).

Typically, firefighter clothing is evaluated on a standing manikin for its thermal properties and comfort using sweating thermal manikins (ISO15831:^2004^, ASTM F1291-16, ASTM F2370-16), or for its protective performance using a flame manikin (ISO13506-1:^2017^). These methods provide, in principle, overall values reflecting the clothing performance, where the heat transfer of individual parts is lumped together. In the standing position, this approach would yield a value representative not only for the overall performance but also locally for individual body parts, since the air layers in the standing position are relatively high (over 20 mm) and their thermal characteristics will be similar for individual body parts. In other studied body postures, the local differences are distinctly larger, where local air gaps can be as low as 4 mm (lower back and posterior thigh in hose-holding posture) and several body parts show air gaps below 15 mm, where their thermal characteristics vary strongly according to the air gap thickness. In such a case, overall clothing characteristics may not be a sufficient parameter to describe its performance since they will not capture the local weakest spots. Therefore, the research effort for better protection of fire fighters should not only be focused on protective fabrics but also on clothing design and associated air gaps in relevant body postures during different firefighting tasks. These new considerations will add an additional dimension to the assessment and the development of future improved fire-fighter protective clothing. For example, thermal manikin data related to garment thermal properties should be supplemented with projection of these properties over range of typical and extreme temperatures possible during the exposure when wearing the garment. This consideration will allow a more realistic estimation of the heat burden on the human body and corresponding classification of protective clothing. Knowing weak points of the protective garment in different scenarios will help a better estimation of a potential burn and heat illness risk, and possibly an application of preventive measures.

## Conclusions

In this study, we investigated differences in the air layers distribution in firefighters' clothing in three postures reflecting realistic on-duty exposure conditions (crawling, hose-holding) and standing upright used in laboratory tests. The body posture induced substantial changes in the air gap thickness on the upper body (chest and back) and lower body (upper and lower leg). These changes were reflected in both the calculated thermal and evaporative resistance of the ensemble, and consequently, in their potential thermal performance in the field. Therefore, it is recommended to consider body postures (and air gap distribution in firefighter clothing) during the evaluation of its protective performance. Secondly, the knowledge of local clothing properties in real-life exposure provides a true protection mapping and gives design inputs to improve local protective properties of firefighters' clothing. This study could also lead towards the development of new standards for evaluating the performance of protective clothing under different postures.
